# Effect of oestrus synchronisation through ovulation delay by vaccination against foot‐and‐mouth disease in Hanwoo (*Bos taurus* coreanae) cows

**DOI:** 10.1002/vms3.1074

**Published:** 2023-01-12

**Authors:** Daehyun Kim, Woo‐Sung Kwon, Jaejung Ha, Jaejo Kim, Danil Kim, Wonyou Lee, Joonho Moon, Junkoo Yi

**Affiliations:** ^1^ Department of Korean Cattle Reserch Livestock Research Institute Gyeongsangbuk‐do Korea; ^2^ Department of Animal Science and Biotechnology Kyungpook National University Gyeongsangbuk‐do Korea; ^3^ Department of Animal and Plant Hygiene Research Animal and Plant Quarantine Agency Gyeongsangbuk‐do Korea; ^4^ Department of Designed Animal and Transplantation Research Seoul National University Seoul Korea; ^5^ Department of Trangenic Cow Generation Lartbio Co., Ltd. Seoul Korea; ^6^ Department of Transgenic Pig Generation GenNBio Co., Ltd. Gyeonggi‐do Korea

**Keywords:** conception rate, foot‐and‐mouth disease vaccine, gonadotropin‐releasing hormone, Hanwoo (*Bos taurus* coreanae) cow, ovulation delay

## Abstract

**Background:**

In Korean cattle, after foot‐and‐mouth disease (FMD) vaccination, anovulation increases, acute immune response is stimulated.

**Objective:**

Here, we aimed to improve the fertility rate by ovulation delay caused by the foot‐and‐mouth disease vaccine.

**Methods:**

160 cows (control, FMD, FMD+Gn250 and FMD+Gn500 groups, with 40 cows each) were used. We analysed the ovulation delay, ovulation rate, conception rate and acute‐phase immune responses.

**Results:**

In the group vaccinated only with FMD, the average follicle size was maintained at 12 mm and ovulation was delayed. The ovulation rate of the FMD+Gn500 group (500 μg gonadotropin‐releasing hormone (GnRH) injections 3 days after the FMD vaccination) was the highest at 81.8%. The ovulation rate of the FMD+Gn250 group (250 μg GnRH injections 3 days after FMD vaccination) was 54.5%, and that of the control group (not FMD vaccinated) was 53.3%. The conception rate was 52.5% (19/40) in the control group, 37.5% (15/40) in the FMD+Gn250 group, and 67.5% (27/40) in the FMD+Gn500 group. Analysis of acute‐phase immune response revealed that the plasma contents of haptoglobin and serum amyloid A increased up to 7 days after vaccination against FMD in all the experimental groups, except the control group.

**Conclusions:**

We concluded that ovulation delay can be employed to improve conception rate after FMD vaccination through a modified ovulation synchronisation method with GnRH.

## Graphical abstract

The purpose of this study is to improve the fertility rate by ovulation delay caused by foot‐and‐mouth disease vaccination. We figure out that ovulation delay can be employed to improve conception rate after FMD vaccination through a modified ovulation synchronization method with 500 μg of GnRH administration.

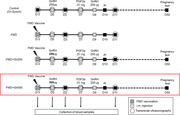

## 1 INTRODUCTION

Many countries across America, Europe and Asia have recently recommended that regular foot‐and‐mouth disease (FMD) vaccinations on farms is the only way to prevent FMD (Grubman & Baxt, 2004; Kahn et al., 2002; Park et al., 2013; Rodriguez & Grubman, 2009). However, previously, we reported that FMD vaccination is accompanied by side effects such as delayed ovulation, early embryo loss, sperm infertility, decreased milk production, increased acute immune response as well as increased body temperature, leading to miscarriage (Ferreira et al., 2016; D. Kim, Ha et al., 2021; D. Kim et al., 2021; Perumal et al., 2013; Yeruham et al., 2001).

Among the various side effects of FMD vaccination, delayed ovulation is associated with fertility rates. Pregnancy through artificial insemination requires semen to be injected into the uterus when an oocyte is ovulated (Bo et al., 2011). However, previous studies have confirmed that FMD vaccination disrupts ovulation by delaying it (D. Kim, Moon et al., 2021).

The mechanism underlying FMD vaccination‐induced ovulation delay is in line with Hansen's inflammation pathways by which infection in the mammary gland (Hansen et al., 2004). According to Hansen's study, it was reported that inflammation leads to anovulation by increasing cytokines and prostaglandin F2α (PGF2α). (Hansen et al., 2004; Peter et al., 1989; Skarzynski et al., 2000; Suzuki et al., 2001). FMD vaccination aggravates the inflammatory response and activates the acute phase response (Hansen et al., 2004; Rodriguez & Grubman, 2009). An increase in acute‐phase immune proteins such as haptoglobin and serum amyloid A inhibits the action of PGF2α and gonadotropin‐releasing hormone (GnRH), leading to ovulation delay (Hansen et al., 2004; D. Kim, Moon et al., 2021; Kujjo et al., 1995; Suzuki et al., 2001). However, although FMD vaccination decreases conception rate due to ovulation delay and early embryo loss, it is essential to prevent FMD in cows (Ferreira et al., 2016; D. Kim, Moon et al., 2021). Therefore, we aimed to make use of the side effects of FMD vaccination for ovulation synchronisation.

The primary goal of this study was to develop OvSynch, a new synchronisation program by grafting ovulation delay following FMD vaccination using GnRH and PGF2α. The principle of OvSynch is to induce ovulation through GnRH injection on day 0 at a random stage, PGF2α injection on day 7, second GnRH injection on day 9, and artificial insemination on day 10 (Geary et al., 1998; Moreira et al., 2000; Pursley et al., 1998).

Delayed ovulation after immunisation against FMD adversely affects fertility. However, no studies have been conducted on improved fertility rates due to FMDV. Therefore, in this study, the ovulation rate and fertility rate when the GnRH‐based OvSynch method is applied after FMDV are comparatively analysed, and a method to improve the decrease in fertility due to FMDV by controlling the amount of GnRH is to be developed.

## 2 MATERIAL AND METHODS

### 2.1 Animals

For this study, 160 cows (four groups, with 40 cows, each) from the Gyeongsangbuk‐do Livestock Research Institute were used. The cows were housed in a stanchion barn with sufficient space during the experiment and fed according to the Korean feeding standard program. Rice straw, mineral blocks, and water were provided ad libitum.

### 2.2 Experimental design

#### 2.2.1 Control group

The OvSynch group received 250 μg GnRH (Gonadon, gonadorelin acetate 100 μg/ml; Dong Bang Co., Seoul, South Korea) intramuscular (i.m.) injection at day 0 at random stage, 25 mg PGF2α (Lutalyse, dinoprost tromethamine 5 mg/ml; Zoetis, Fairfield, NJ, USA) i.m. injection on day 7, a second 250 μg GnRH i.m. injection on day 9, and artificial insemination on day 10 is hereinafter referred to as the ‘control’. The control group was artificially inseminated using OvSynch.

#### 2.2.2 FMD group

Only the FMD vaccination group received a 50% protective dose i.m. injection of the FMD vaccine (Bioaftogen, FMDV vaccine O1 Campos, A24 Cruzeiro and A2001 Argentina; Biogénesis Bagó, Buenos Aires, Argentina) and is hereinafter referred to as ‘FMD’. In the FMD group, an ovulation delay was confirmed through transrectal ultrasonography after FMD vaccination, and artificial insemination was not performed.

#### 2.2.3 FMD+Gn250 group

The FMD+Gn250 group received a 50% protective dose i.m. injection of the FMD vaccine, 250 μg GnRH (Gonadon, gonadorelin acetate 100 μg/ml; Dong Bang Co., Seoul, South Korea) i.m. injection 3 days after FMD vaccination, 25 mg PGF2α i.m. injection on day 7, a second 250 μg GnRH i.m. injection on day 9, and artificial insemination on day 10.

#### 2.2.4 FMD+Gn500 group

The FMD+Gn500 group received 50% protective dose i.m. injection of the FMD vaccine, 500 μg GnRH i.m. injection 3 days after FMD vaccination, 25 mg PGF2α i.m. injection on day 7, a second 250 μg GnRH i.m. injection on day 9, and artificial insemination on day 10.

### 2.3 Ovulation and pregnancy test

**FIGURE 1:**
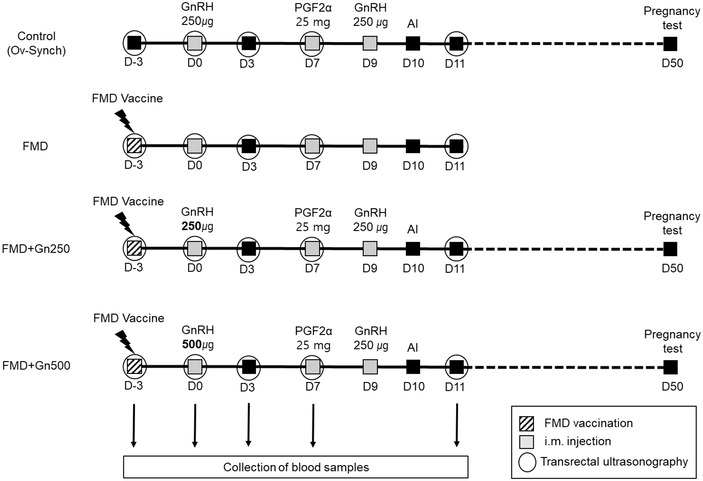
Schematic diagram of the experimental design. The diagonal square is the time of FMD vaccination, grey square is the time of i.m. injection, and circle is the time of ovulation test confirmed through transrectal ultrasonography. Blood collection was performed on day –3, 0, 3, 7 and 11.

Ovulation tests were performed using transrectal ultrasonography (DRAMIŃSKI – ED2; DRAMIŃSKI, Gietrzwałd, Poland) on day –3, 0, 3, 7 and 11 (Figure 1). Pregnancy was confirmed in the control, FMD+Gn250, and FMD+Gn500 groups 40 days after artificial insemination. Pregnancy tests were performed using transrectal ultrasonography (HS‐101 V; Honda, Tokyo, Japan) on day 50 (Figure 1).

### 2.4 Plasma collection and concentration of haptoglobin and serum amyloid A(SAA)

Blood samples were collected from all groups on day –3, 0, 3, 7 and 11 (Figure 1). A detailed method for blood sample collection and analysis using a bovine enzyme linked immunosorbent assay (ELISA) kit (haptoglobin, SAA) has been reported by Kim et al. (D. Kim, Moon et al., 2021). Plasma haptoglobin levels were measured using colorimetric haptoglobin ELISA kit (Catalogue No. E‐10HPT; ICL Inc., Portland, OR, USA), and plasma SAA levels were measured using SAA ELISA kit (Catalogue No. TP 802; Tridelta Development Ltd., Kildare, Ireland). ELISA analysis was performed through an ELISA reader (Gen5; BioTek, Seoul, Korea) at 450 nm absorbance.

### 2.5 Statistical analysis

All statistical analyses were performed using GraphPad Prism (version 8.0.1; GraphPad Software Inc., La Jolla, CA, USA). Differences in plasma haptoglobin and SAA concentrations were analysed using two‐way analysis of variance (ANOVA) (Tukey's multiple comparisons test), and the conception rate was analysed using the chi‐square test. Statistical significance was set at *p* < 0.05.

## 3 RESULTS

### 3.1 Ovulation rate and follicle size

**FIGURE 2:**
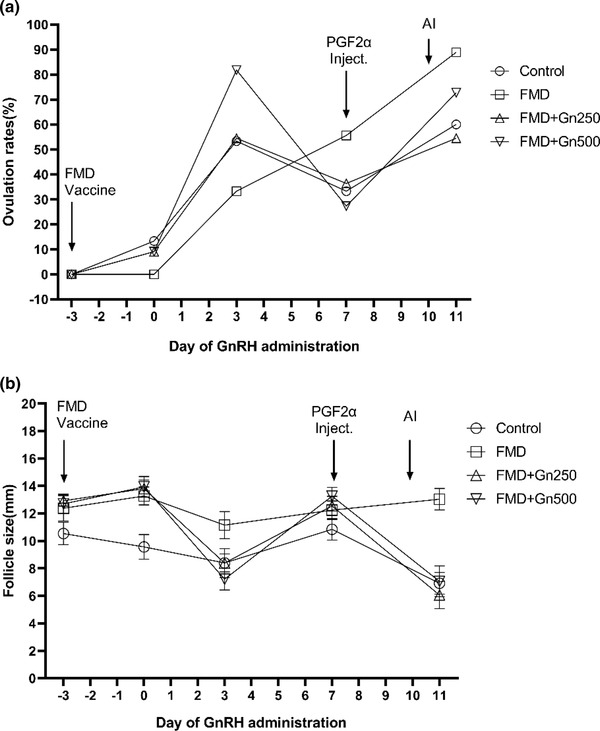
Change in ovulation rate and follicle size depending on the different treatments (*n* = 160). (a) Change in ovulation rate by treatment. The black line connected by ‘○’ represents the mean ovulation rate of the control group (NOT vaccinated). The black line connected by ‘□’ represents the mean ovulation rate of the FMD group (Only vaccinated). The black line connected by ‘△’ represents the mean ovulation rate of the FMD+Gn250 group (250 μg GnRH i.m. injection on day 0). The black line connected by ‘▽’ represents the mean ovulation rate of the FMD+Gn500 group (500 μg GnRH i.m. injection on day 0). (b) Change of follicle size by treatment. The black line connected by ‘○’ represents the mean follicle size of the control group (Not vaccinated). The black line connected by ‘□’ represents the mean follicle size of the FMD group (Only vaccinated). The black line connected by ‘△’ represents the mean follicle size of the FMD+Gn250 group (250 μg GnRH i.m. injection at day 0). The black line connected by ‘▽’ represents the mean follicle size of the FMD+Gn500 group (500 μg GnRH i.m. injection at day 0). FMD, FMD+Gn250 and FMD+Gn500 group was injected FMD vaccine at day –3 (except for the control group). The control, FMD+Gn250 and FMD+Gn500 groups were injected with 25 mg PGF2α on day 7, injected with 250 μg GnRH on day 9 and artificial insemination was conducted on day 10. Blood samples were collected on days –3, 0, 3, 7 and 11.

FMD vaccination affected ovulation. Specifically, ovulation was delayed in three FMD groups (FMD, FMD+Gn250 and FMD+Gn500) that received the FMD vaccination on day 0. The ovulation rate of the FMD+Gn500 group was 81.8%, which was higher than that of the other groups on day 3 (control 53.3%, FMD 33.3 and FMD+Gn250 54.5%) (Figure 2a). The ovulation rate 1 day after artificial insemination was 72.7% in the FMD+Gn500 group, which was higher than that of the other groups (Control, FMD+Gn250) except for FMD group on day 11 (Figure 2a). In the FMD group, because GnRH was not administered, ovulation delay was confirmed after FMD vaccination, and the ovulation delay was completely resolved on day 11 (Figure 2a).

Follicle size showed a pattern similar to synchronisation flow (Figure 2b). The size of the follicles on day 0 was 13.28 ± 0.65 mm in the FMD, 13.82 ± 0.62 mm in the FMD+Gn250 and 13.93 ± 0.75 mm in the FMD+Gn500 groups, markedly higher than that of the control group (9.56 ± 0.90 mm) (*p* < 0.05) (Figure 2b). On the 7th day, when 25 mg PGF2α was injected, the follicle size was 12.24 ± 0.67 mm in the FMD, 12.55 ± 0.70 mm in the FMD+Gn250 and 13.26 ± 0.64 mm in the FMD+Gn500 groups, again markedly higher than that of the control group value (10.84 ± 0.78 mm) (*p* < 0.05) (Figure 2b).

### 3.2 Acute phase immune response and conception rate

**FIGURE 3:**
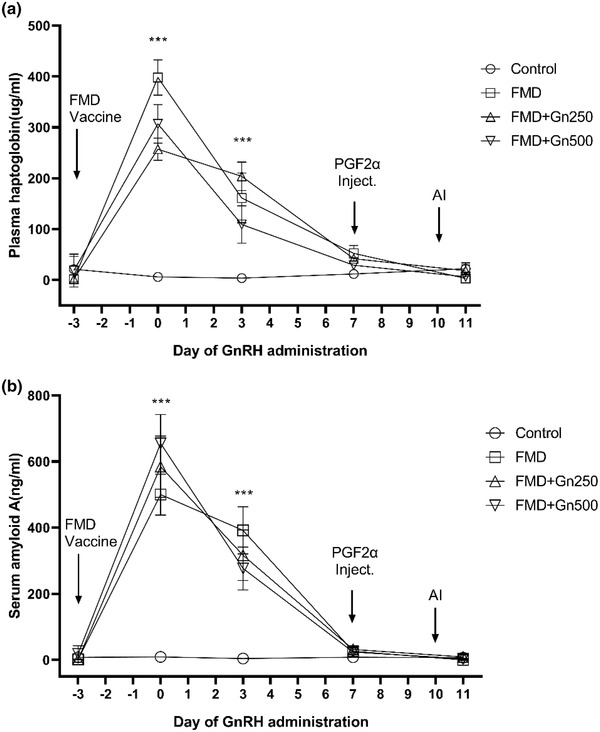
Plasma concentrations of haptoglobin and serum amyloid A depending on the different treatments (*n* = 160). (a) Plasma haptoglobin and (b) serum amyloid A levels. The black line connected by ‘○’ represents the mean ovulation rate of the control group (NOT vaccinated). The black line connected by ‘□’ represents the mean ovulation rate of the FMD group (Only vaccinated). The black line connected by ‘△’ represents the mean ovulation rate of the FMD+Gn250 group (250 μg GnRH i.m. injection on day 0). The black line connected by ‘▽’ represents the mean ovulation rate of the FMD+Gn500 group (500 μg GnRH i.m. injection on day 0). The FMD, FMD+Gn250 and FMD+Gn500 groups were injected with the FMD vaccine on day –3 (except for the control group). The control, FMD+Gn250 and FMD+Gn500 groups were injected with 25 mg of PGF2α on day 7, injected with 250 μg GnRH on day 9 and artificial insemination on day 10. Blood collection was performed on day –3, 0, 3, 7 and 11. ***Significance level *p* < 0.001.

The analysis of the concentrations of haptoglobin and SAA, representing the acute phase immune response after FMD vaccination, confirmed that it dramatically increased up to 7 days after FMD vaccination (*p* < 0.001) (Figure 3). All vaccination groups (FMD, FMD+Gn250 and FMD+Gn500), except the control group, showed increased levels of haptoglobin and SAA after 3 days of FMD vaccination (*p* < 0.001) (Figure 3). However, at the time of artificial insemination (day 10), haptoglobin and SAA levels recovered to the basal level in all experimental groups (Figure 3). The pregnancy rate in the FMD+Gn500 group was significantly higher than that in the FMD+Gn250 group (*p* < 0.05) and we observed no adverse effect on the pregnancy rate compared to the control group (Table 1).

**TABLE 1:** Pregnancy rates and timing following different treatments (*n* = 120)

Group	No. of pregnant cows	Total	Pregnancy rates (%)
Control	21	40	52.5%^a^
FMD+Gn250	15	40	37.5%^b^
FMD+Gn500	27	40	67.5%^a^
Total	63	120	52.5%

*Note*: Values with different superscript letters, a and b, are significantly different at *p* < 0.05.

## 4 DISCUSSION

Various oestrus and ovulation synchronisation programs have been developed to improve the fertility rate, including OvSynch of GnRH and PGF2α bases, progesterone‐releasing intravaginal devices (PRID), and J‐synch methods using oestrogen and progesterone ratio (Bo et al., 2019; Geary et al., 1998; Moreira et al., 2000; Perez‐Mora et al., 2020; Pursley et al., 1998). A method of combining PRID and OvSynch was developed to compensate for the shortcomings of OvSync (Geary et al., 1998; Moreira et al., 2000; Pursley et al., 1998).

Furthermore, Bo et al. recently developed a J‐synch method that maximises the efficiency of ovulation by applying equine chorionic gonadotrophin (eCG) to the method of combining progesterone‐releasing intravaginal devices (PRID) and OvSynch (Bo et al., 2019, 2011; Perez‐Mora et al., 2020; Pessoa et al., 2016). In addition, a study was conducted on how to increase the reproduction rate and efficiency of large‐scale farms through fixed‐time artificial insemination (FTAI) or fixed‐time embryo transfer (FTET) methods based on J‐synch (Baruselli et al., 2011; Bo & Baruselli, 2014; Bo et al., 2019, 2011; Pereira et al., 2016).

However, the final goal of these oestrus and ovulation synchronisation methods is to achieve higher conception rate by improving oestrus and ovulation synchronisation rates (Bo et al., 2019; D. H. Kim et al., 2020; Perez‐Mora et al., 2020).

Parity, body condition score, reproductive and nutritional status, and ovulation delay by FMD vaccine in cows are highly associated with the conception rate after artificial insemination and embryo transfer (Bo et al., 2011; D. Kim, Moon et al., 2021; Perez‐Mora et al., 2020; Wu & Zan, 2012). Farm animals must be vaccinated to prevent FMD, but farm profits decrease if the conception rate decreases due to side effects such as ovulation delay and miscarriage of pregnant cows (Ferreira et al., 2016; D. Kim, Ha et al., 2021; D. Kim, Moon et al., 2021).

In this study, FMD vaccination‐induced ovulation delay was designed to be used as a part of ovulation synchronisation, and our research results confirmed the feasibility of this design. The ovulation synchronisation method, which has been improved so far, is a method of increasing the rutting and fertility rate starting at random stage. However, in this study, a unique situation was artificially created through FMD vaccination, and this was applied to ovulation synchronisation.

Furthermore, we have demonstrated previous work that FMD vaccines induce inflammation and delay ovulation through increased acute immune response (D. Kim, Moon et al., 2021). More importantly, it was confirmed that the delayed large follicle was the largest time after 3 days of FMD vaccination, so it was possible to incorporate it into ovulation synchronisation in this study. Therefore, this study used the side effects (ovulation delay) of FMD vaccination to improve conception rate.

The OvSynch is effective when there is a large follicle of >10 mm in the random stage, and it is expected that delaying ovulation through FMD vaccination can lead to a higher oestrous rate. Although not shown in the results, 86.6% (104/120) of cows vaccinated with FMD had delayed ovulation (follicle size >10 mm), which was significantly higher than 69.4% (41/59) of those with large follicles (>10 mm) at the random stage in our previous study (D. H. Kim et al., 2020).

As expected from our hypothesis, injecting GnRH into delayed follicles after FMD vaccination was accompanied by higher ovulation and conception rates compared to the other groups (control and FMD+Gn250). In addition, the increased acute immune response due to FMD vaccination also recovered to the basal level at the time of artificial insemination, and no obvious inconsistencies were noticed with pregnancy maintenance and parturition after the pregnancy test.

Ferreira et al. reported an increase in early embryonic loss as a result of FMD vaccination at the age of 30 and 90 days of pregnancy, respectively, and reported the results of a study on haptoglobin in plasma and increased rectal temperature (el‐Belely et al., 1994; Ferreira et al., 2016). New ovulation synchronisation methods developed through the results of this study are likely to have a negative effect on pregnancy. However, in a previous study, we checked concentration of haptoglobin and SAA in plasma, and ruminoreticular temperature for 16 days longer than in a study by Ferreira et al. and found that it recovered to a normal range about 10 days after FMD vaccination (Ferreira et al., 2016; D. Kim, Moon et al., 2021).

There are no studies applied to ovulation synchronisation using the side effects such as anovulation of the FMD vaccine. Since FMD vaccination combined with OvSynch can achieve both vaccination and improve the conception rate, this method not only prevents cows from suffering from FMD, it also helps to improve the profit of farms through FMD vaccination.

In conclusion, ovulation delay and decreased conception rate are characteristic side effects of FMD vaccination; however, we focused on using these shortcomings to derive positive effects. By administering twice the amount of GnRH at the time of delay owing to FMD vaccination, oestrous synchronisation became more precise. The method of reverse utilising ovulation delay due to FMD vaccines may help improve the conception rate and consequently financially benefit farmers.

## AUTHOR CONTRIBUTIONS

Conceptualisation: D.K., W.‐S.K., J.M. and J.Y. Methodology: D.K., W.‐S.K., J.K., Di.K., and W.L. Software: J.M., J.H. and W.‐S.K. Validation: D.K., J.H., J.K., Di.K. and W.L. Formal analysis: J.M. and W.‐S.K. Investigation: D.K., J.H., J.K., Di.K. and W.L. Data curation: J.M., W.L. and W.‐S.K. Writing – original draft preparation: D.K. and W‐S.K. Writing – review and editing: J.M. and J. Y. Visualisation: D.K. and J.H. Supervision: J.M. and J.Y. All the authors have read and agreed to the published version of the manuscript.

## CONFLICT OF INTEREST

We confirm that this manuscript has not been published in whole or in part and is not being considered for publication elsewhere. There are no any conflict of interest for all authors.

## CONSENT FOR PUBLICATION

All authors have consented to the submission of this manuscript for publication.

### ETHICS STATEMENT

This study was conducted in accordance with the Declaration of Helsinki and approved by the Institutional Animal Care and Use Committee (IACUC) of the Gyeongsangbuk‐do Livestock Research Institute, Yeongju, Korea (protocol code GAEC/127/20 approved on December 15, 2020).

### PEER REVIEW

The peer review history for this article is available at https://publons.com/publon/10.1002/vms3.1074.

## Data Availability

The data that support the findings of this study are available from the corresponding author upon reasonable request.
